# An SNP-based saturated genetic map and QTL analysis of fruit-related traits in cucumber using specific-length amplified fragment (SLAF) sequencing

**DOI:** 10.1186/1471-2164-15-1158

**Published:** 2014-12-22

**Authors:** Qingzhen Wei, Yunzhu Wang, Xiaodong Qin, Yunxia Zhang, Zhentao Zhang, Jing Wang, Ji Li, Qunfeng Lou, Jinfeng Chen

**Affiliations:** State Key Laboratory of Crop Genetics and Germplasm Enhancement, College of Horticulture, Nanjing Agricultural University, Weigang Street No.1, Nanjing, 210095 China

**Keywords:** SLAF-seq, Genetic map, SNP, *Cucumis sativus* L, QTL analysis

## Abstract

**Background:**

Cucumber, *Cucumis sativus* L., is an economically important vegetable crop which is processed or consumed fresh worldwide. However, the narrow genetic base in cucumber makes it difficult for constructing high-density genetic maps. The development of massively parallel genotyping methods and next-generation sequencing (NGS) technologies provides an excellent opportunity for developing single nucleotide polymorphisms (SNPs) for linkage map construction and QTL analysis of horticultural traits. Specific-length amplified fragment sequencing (SLAF-seq) is a recent marker development technology that allows large-scale SNP discovery and genotyping at a reasonable cost. In this study, we constructed a high-density SNP map for cucumber using SLAF-seq and detected fruit-related QTLs.

**Results:**

An F_2_ population of 148 individuals was developed from an intra-varietal cross between CC3 and NC76. Genomic DNAs extracted from two parents and 148 F_2_ individuals were subjected to high-throughput sequencing and SLAF library construction. A total of 10.76 Gb raw data and 75,024,043 pair-end reads were generated to develop 52,684 high-quality SLAFs, out of which 5,044 were polymorphic. 4,817 SLAFs were encoded and grouped into different segregation patterns. A high-resolution genetic map containing 1,800 SNPs was constructed for cucumber spanning 890.79 cM. The average distance between adjacent markers was 0.50 cM. 183 scaffolds were anchored to the SNP-based genetic map covering 46% (168.9 Mb) of the cucumber genome (367 Mb). Nine QTLs for fruit length and weight were detected, a QTL designated *fl3.2* explained 44.60% of the phenotypic variance. Alignment of the SNP markers to draft genome scaffolds revealed two mis-assembled scaffolds that were validated by fluorescence *in situ* hybridization (FISH).

**Conclusions:**

We report herein the development of evenly dispersed SNPs across cucumber genome, and for the first time an SNP-based saturated linkage map. This 1,800-locus map would likely facilitate genetic mapping of complex QTL loci controlling fruit yield, and the orientation of draft genome scaffolds.

**Electronic supplementary material:**

The online version of this article (doi:10.1186/1471-2164-15-1158) contains supplementary material, which is available to authorized users.

## Background

Cucumber (*Cucumis sativus* L., 2n = 2x = 14) is one of the most important vegetable crops cultivated worldwide, immature fruits of which are consumed cooked, processed, or fresh in a considerable amount. Agricultural production of cucumbers and gherkins accounted for more than 2 million hectares of land yielding 62 million tons of produce in 2010 (http://faostat3.fao.org). However, cucumber has a very narrow genetic base and lack of molecular polymorphism [1–4], which impedes the construction of saturated genetic maps and map-based cloning of horticultural important genes. In the past decades, cucumber linkage maps were mostly composed of dominant markers [i.e. random amplified polymorphic DNAs (RAPDs), and amplified fragment length polymorphisms (AFLPs)], and did not reach saturated (average marker distance less than 2 cM) due to insufficient marker number [5–9].

Draft genome assemblies of three cucumber lines (9930, Gy14, B10) [10–12] successively published provide a good opportunity for developing simple sequence repeats (SSRs) as co-dominant markers in map construction [13]. Several SSR-based maps have been developed with 100 ~ 300 or even more markers [11, 14–19], and genes controlling cucumber scab resistant (*Ccu*), compact growth (*cp*), Zucchini yellow mosaic virus resistance (*zym*) , uniform immature fruit color (*u*), tuberculate fruit (*Tu*), spine color and mature fruit color (*B*), and dull skin (*D*) were mapped or fine mapped [4, 17, 20–24]. The inter-subspecific genetic map with 995 SSRs constructed by Ren *et al.* [14] is the most saturated, followed by an intra-varietal map containing 735 loci [11]. Unfortunately, over one quarter of the mapped SSRs in this inter-subspecific map were found clustering in chromosomes 3, 4, 5, 6, and 7 due to the small mapping population [77 recombinant inbred lines(RILs)] and possible chromosomal rearrangements between the two parents (cultivated cucumber Gy14 and the wild *C. sativus* var. *hardwickii* PI 183967). Two consensus maps were developed in cucumber to increase marker density, which were constructed by Zhang *et al.* (1369 marker loci) [25], and Yang *et al.* (1681 marker loci), respectively [26]. Both of them employed the Gy14 × PI 183967 map with 995 SSRs for map integration [16], whereas marker orders in recombination suppression regions in the 1369-point map were not well placed. The 1681-locus consensus map overcame this drawback by the integrating intra-varietal map by Yang *et al.* [11], and improved marker orders and density in three chromosomes particularly in chromosome 4. Despite the high marker density in consensus maps, it is still difficult to construct saturated maps for F2 or RIL populations derived from intra-varietal crosses to conduct QTL analysis and molecular mapping in certain populations.

Single-nucleotide polymorphisms (SNPs) are the most abundant and stabile form of genetic variation in most genomes, which have become the marker type of choice in many evolutionary and ecological studies [27–29]. The advent of massive parallel next-generation sequencing (NGS) technologies has made it possible for high-throughput identification and genotyping of SNPs. However, whole-genome deep re-sequencing is still cost-prohibitive for sequencing and genotyping large populations and usually not necessary [30]. Reduced representation library (RRL) sequencing is one strategy to bring down the cost through genome reduction [25, 31, 32]. Restriction-site associated DNA sequencing (RAD-seq) reduces genome complexity by sequencing only the DNA fragments with restriction sites in spite of length, and has been proven to be a useful tool for SNP discovery and genetic mapping [33–35]. 2b-RAD is a streamlined RAD approach that sequences uniform fragments generated by type IIB restriction endonuclease, which is suitable for species with large genomes including humans [17]. Recently, specific-length amplified fragments sequencing (SLAF-seq) was developed as a modified RRL sequencing strategy for *de novo* SNP discovery and genotyping of large populations [36], which has generated high-density genetic maps with abundant SNPs for common carp, sesame and soybean [36–38].

Studies have shown that a number of fruit-related traits are controlled by quantitative trait loci (QTLs) in cucumber, such as fruit weight and fruit shape index (i.e., fruit length, diameter, length/diameter ratio and length/stalk ratio) [8, 39–45]. Fruit length and weight are two traits that significantly correlate with yield and commercial quality of the cultivated cucumber. With a SRAP (sequence-related amplified polymorphism)-based genetic map (257 loci), Yuan *et al.* [40] identified five QTLs for fruit weight and seven QTLs for fruit length. More recently, Cheng *et al.* [42] identified five QTLs for immature fruit length using 234 SSRs on LGs 1, 4, and 6. Miao *et al.* [43] detected three QTLs for immature fruit length on LG5 and LG6 with a previously constructed map (245 SSRs). Five QTLs conditioning fruit weight were also identified on LG1 and LG5 [41]. However, the information is still insufficient for map-based gene cloning for cucumber yield and fruit quality improvement.

In this study, a high-throughput and cost-effective SLAF-seq approach was employed to generate an SNP-based genetic map for cucumber, which contained 1,800 high quality SNPs and spanned 890.79 cM with an average marker interval of 0.50 cM. Physically, we anchored 183 of the ‘9930’ draft genome scaffolds with 168.9 Mb sequences. Two mis-assembled scaffolds were verified. Nine QTLs controlling fruit length and weight were detected on four linkage groups.

## Results

### High-throughput SLAF sequencing and genotyping

**Figure 1:**
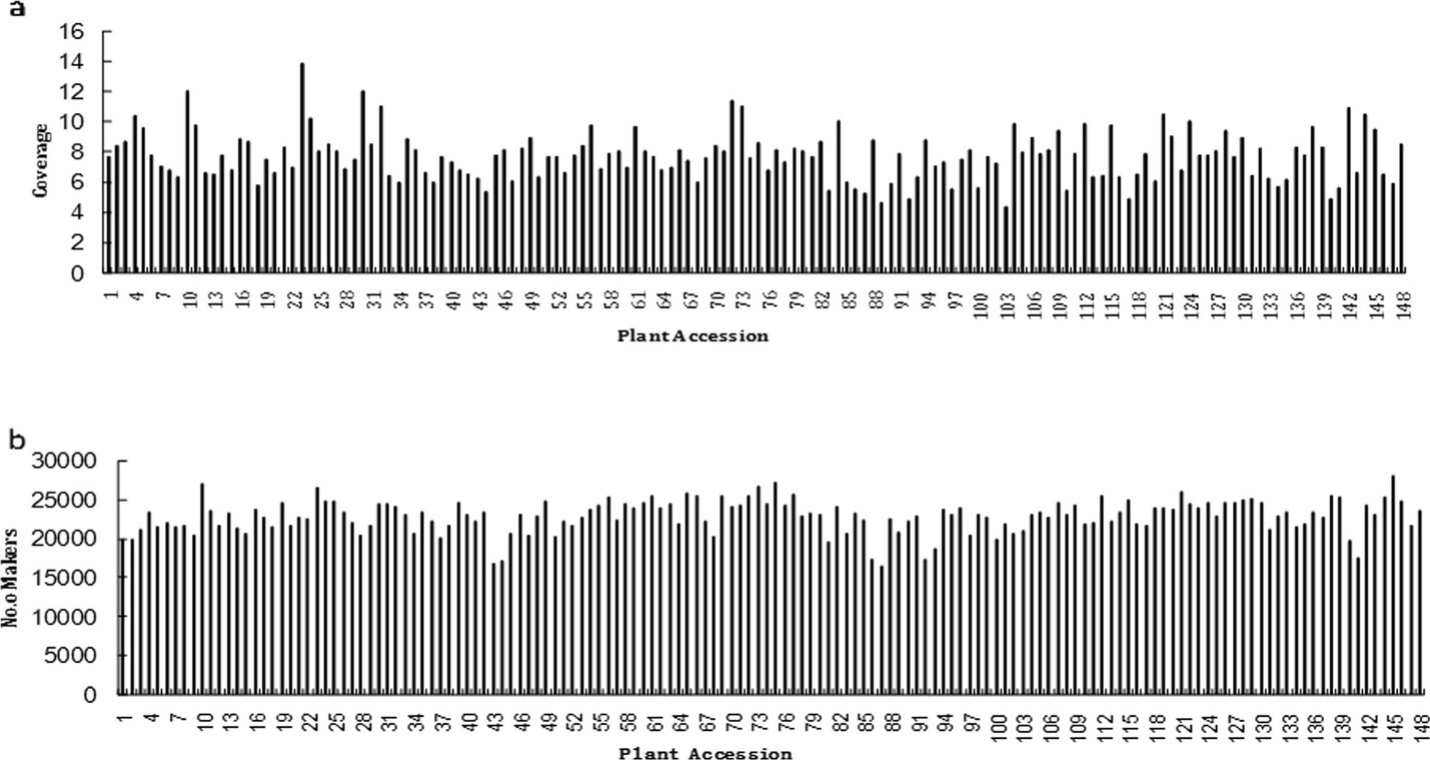
**Coverage and number of markers for each of the F**_**2**_ **individuals.** The x-axes in both **a** and **b** indicate each of the F_2_ individuals; the y-axe in a indicates marker coverage, and the y-axe in **b** indicates the number of markers developed for each F_2_ plant.

A total of 10.76 Gb raw data was generated from Illumina sequencing and SLAF library construction, which contained 75,024,043 pair-end reads with a length of 100 bp. The GC (guanine-cytosine) content was 46.29%, and Q20 ratio (a quality score of 20) was 83.78%. In the paternal inbred line (NC76), 2,555,002 reads and 32,121 SLAFs were generated, with an average coverage of 79.54-fold for each SLAF. In the maternal line (CC3), the number of reads produced for 31,898 SLAFs was 3,050,383, and the average cover for each SLAF marker was 95.63-fold. For the analysis of the F_2_ mapping population, 84,900 to 367,251 reads were generated for the development of 16,497 to 28,155 SLAF markers for each plant; the marker coverage ranged from 4.34 to 13.81-fold, with an average of 7.74-fold (Figure 1a). The average count of SLAFs per individual was 22,866 (Figure 1b, distribution of DNA fragments digested by enzyme on the genome is presented in Additional file 1: Figure S5).

**Figure 2:**
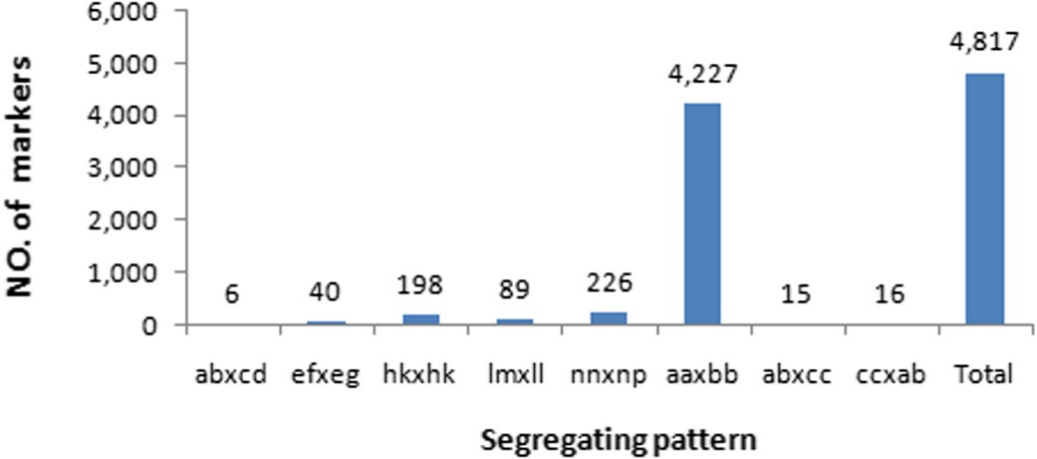
**Number of markers for each segregation pattern.**

After correcting or discarding low-depth SLAF tags, 52,684 high-quality SLAFs were identified, among which 5,044 were polymorphic with a polymorphism rate of 9.57% (Table 1, Additional file 2: Table S1). The parental lines were given with different alphabets as genotypes to determine segregation patterns, and 4,817 from the 5,044 polymorphic SLAFs were successfully encoded and grouped into eight segregation patterns (ab × cd, ef × eg, hk × hk, lm × ll, nn × np, aa × bb, ab × cc and cc × ab) following a genotype encoding rule (Figure 2; Additional file 2: Table S4). Since the two parents (NC76 and CC3) are homozygous cucumber inbred lines with genotypes of aa and bb, only the 4,227 markers that fell into the aa × bb segregation pattern were used in linkage analysis (Figure 2).

**Table 1:** **Discovery of SLAF markers**

Type	Number of SLAF markers	Number of reads	Ratio
Polymorphism SLAF	5,044	4,085,646	9.56%
Non-polymorphism SLAF	47,640	28,164,020	90.44%
Total	52,684	32,249,666	100.00%

### Genetic linkage map

After removing incomplete and significant segregation distortion markers, 1,800 SNPs were retained for genetic map construction. JoinMap 4.0 assigned all the 1,800 markers to seven LGs of cucumber (Table 2, Additional file 3). The average integrity of the mapped SNP markers reached 94.74%, representing a relatively high map quality. Details of this SNP-based cucumber map are presented in Additional file 4 and summarized in Table 2.

**Table 2:** **Summary of the SNP-based cucumber genetic map**

Linkage group ID	No. of mapped SNPs	Map length(cM)	Marker interval(cM)	Gaps < =5	scaffolds anchored	Physical length (Mb)
1	321	136.48	0.43	100.00%	36	25.93
2	252	131.20	0.52	100.00%	26	20.66
3	344	193.65	0.56	99.13%	32	34.25
4	199	104.89	0.53	100.00%	26	19.13
5	312	139.14	0.45	100.00%	24	24.97
6	206	119.31	0.58	100.00%	21	27.18
7	166	66.12	0.40	100.00%	18	16.76
Total	1,800	890.79	0.50	100.00%	183	168.88

The total genetic length of the SNP map was 890.79 cM in seven linkage groups with a mean marker distance of 0.5 cM between adjacent markers. The largest linkage group (LG3) contained 344 SNPs, while the smallest LG7 had 166 SNPs. On average, there were 257 SNP markers in each linkage group. The genetic distances of seven linkage groups spanned 66.12 cM (LG7) ~ 193.65 cM (LG3), with mean marker intervals ranged from 0.40 cM to 0.58 cM. ‘Gap < = 5′ value, which reflected linkage degree among markers, were 100.00% on all linkage groups except for LG3 (99.13%).

### Validation of the SNP-based genetic map

The quality of this genetic map was evaluated by heat maps which directly reflected recombination relationships among markers in seven linkage groups (Additional file 5). Each cell represented a recombination rate between two adjacent markers, the level of which was visualized by different colors ranging from yellow to purple (yellow indicated a lower recombination rate; purple indicated a higher rate). Heat maps indicated SNP markers in most LGs were well ordered.

**Figure 3:**
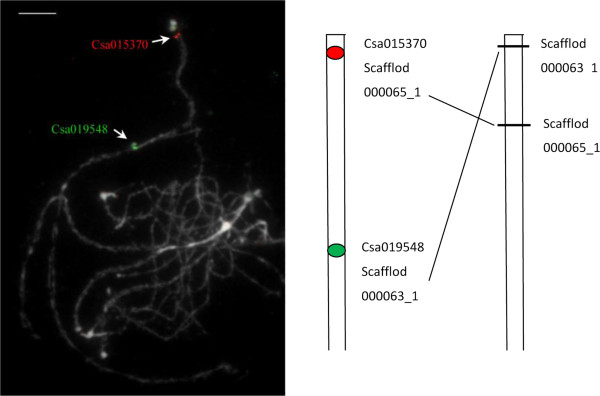
**Two scaffold mis-assemblies revealed by single-copy gene FISH. a** Locations of two single-copy genes Csa015370 (scaffold000063_5, red) and Csa019548 (scaffold000063_1, green) on cucumber pachytene chromosome spreads were indicated with arrows.Scale bar = 5 μm. **b** Ideogram showing the physical positions of two scaffolds revealed by FISH and in the ‘9930’ draft genome assemblies.

All the mapped SNPs were used to anchor and orient scaffolds of ‘9930’ draft genome assemblies. Physically, 183 scaffolds were anchored onto the SNP map covering 69.00% (168.9 Mb) of the ‘9930’ draft genome sequences (Table 2). Only 44 of the 1,800 SNP markers did not have BLAST hits. Locations of all mapped loci on the SNP linkage map in the‘9930’ draft genome assembly Version 2.0 are provided in Additional file 4. For most scaffolds, more than one SNP was assigned to the same scaffold (~10 SNPs per scaffold). In several cases, the order of scaffolds oriented by the genetic map disagreed with that in the draft genome assembly. For example, we anchored scaffold000063_5 and scaffold000063_1 to the distal end and one third region of chromosome 5 respectively, whereas their positions in the genome assemblies were ~ 3 Mb and ~ 0.3 Mb, respectively. We thereby designed two single-copy gene probes (Csa015370 from scaffold000063_5, and Csa019548 from scaffold000063_1) (see Methods) to verify their positions by fluorescence *in situ* hybridization (FISH). FISH images showed that scaffold000063_5 was at the distal end on chromosome 5 and scaffold000063_1 were in a one third region (~10 Mb) on the same chromosome. Thus these two scaffolds were mis-assembled (Figure 3).

### QTL mapping for fruit length and weight

Phenotypic data (including family means, standard errors and distribution of fruit length and weight) in both F2 and F2:3 families are presented in Additional file 6. Nine QTLs were detected for fruit length (*fl* for immature fruit length, and *mfl* for mature fruit length) and fruit weight (*fw* for immature fruit weight) (Table 3). Immature fruit length had four QTLs, and the most prominent QTL designated *fl3.2*, accounted for 44.60% of the observed phenotypic variance (Additional file 1: Figure S2). Twelve SNPs covered this interval (Table 3 and Additional file 5). A QTL designated *fl3.1* explained 39.10% of phenotypic variance and was also located on LG3. The effects of other QTLs (*fl1.1* and *fl6.1*) were comparatively smaller.

**Table 3:** **Genetic mapping and QTL analysis of cucumber fruit traits** **in F**_**3**_ **populations**

Trait	QTL	LG	Pos. IM (cM)	Closest marker	Start marker	End marker	LOD		% Expl.		No.of SNPs in mapped region
							Minimum	Maximum	Minimum	Maximum	
Fruit length	*fl1.1*	1	45.3-51.6	Maker12120	Maker15971	Maker10660	2.25	3.56	7.60	11.60	15
	*fl3.1*	3	136.4-137.1	Marker5482	Marker17152	Marker5894	14.03	14.32	38.50	39.10	4
	*fl3.2*	3	150.5-155.9	Marker14961	Marker17179	Marker1137	14.42	16.97	39.30	44.60	17
	*fl6.1*	6	57.0-66.7	Marker13991	Marker3014	Marker11267	4.34	5.13	14.00	16.30	25
Mature fruit length	*mfl1.1*	1	45.0-50.3	Marker13691	Marker15971	Marker10158	4.15	5.03	13.60	16.00	11
	*mfl1.2*	1	100.1-103.0	Marker16756	Marker16756	Marker12772	4.55	5.24	14.60	16.60	5
	*mfl3.1*	3	150.5-157.3	Marker7219	Marker17179	Marker1225	12.00	13.32	34.00	36.90	19
Fruit weight	*fw3.1*	3	115.2-117.1	Marker6431	Marker7424	Marker5909	8.31	9.43	25.20	28.30	11
	*fw3.2*	3	146.8-153.5	Marker15161	Marker12129	Marker12274	8.16	9.59	24.60	28.30	12

Three QTLs were detected for mature fruit length, with the largest effect displayed by *mfl3.1*, explaining 36.39% of the observed phenotypic variance (Table 3). Sixty-six SNPs were discovered within the chromosomal region of *mfl3.1. mfl1.1* (Additional file 1: Figure S4) and *mfl1.2*, two QTLs for mature fruit length, were detected on LG1 with maximum LOD scores of 5.03 and 5.46, respectively. Notably, *mfl1.1* and *fl1.1* shared the same start marker (Marker15971), as well as a physical length of 5 cM in the mapped region.

Two QTLs for immature fruit weight (*fw*) were detected, which both explained 28.30% of the observed fruit weight variance at maximum (Additional file 1: Figure S3). QTL *fw3.1* spanned an interval between 115.2 ~ 117.1 cM, where eleven SNPs were identified; the closest marker linked with *fw3.1* was Marker643. The other QTL, *fw3.2*, was flanked by Marker12129 and Marker12274, and twelve SNPs covered this chromosomal region.

## Discussion

### A cost-efficient method of rapid SNP discovery and genotyping using SLAF-seq

NGS-based marker discovery and genotyping technologies provide a good opportunity for developing SNP markers, which are being applied to many studies [46–48]. Whole-genome deep re-sequencing and low coverage sequencing is very costly for large populations and usually unnecessary for linkage mapping and quantitative trait locus mapping. Reduced-representation sequencing offers an approach to sample and sequence a small set of genome regions instead of the whole genome [25, 30]. RAD-seq sequences short DNA fragments with restriction sites that are digested by restriction endonuclease despite the length of those fragments. It has been applied for SNP discovery and linkage map construction in organisms such as stickleback, rainbow trout, barley, and ryegrass [34, 49–51]. 2b-RAD is a streamlined RAD approach that uses type IIB restriction endonuclease to produce uniform fragments sequenced through NGS platforms [17]. It can screen almost every restriction site in the genome and is simpler than existing RAD protocols. However, one drawback of 2b-RAD is the read length (33 ~ 36 bp) constrained by type IIB activity. SLAF-seq is a recently developed enhanced RRL sequencing strategy for *de novo* SNP discovery and genotyping of large populations [36]. Like 2b-RAD, SLAF-seq can also adjust marker identification and genotyping to meet personalized research purposes. The read length in SLAF-seq ranges between 30 ~ 50 bp, which may provide efficient locus discrimination as compared to that in 2b-RAD (33 ~ 36 bp). SLAF-seq was used to construct saturated SNP-based genetic maps in sesame (1,233 SNPs), soybean (5,308 SNPs), and develop 7E chromosome-specific molecular markers for Thinopyrume longatum [37, 38, 52]. In this study, we generated 10.76 Gb raw data, 75,024,043 pair-end reads, developed 32,121 SLAFs through high-throughput SLAF sequencing. 1,800 polymorphic SNPs were identified for linkage map construction.

### The saturated SNP-based genetic linkage map in cucumber

In the present study, we report an SNP-based genetic linkage map in cucumber using SLAF sequencing technology. Due to the narrow genetic base in cucumber, it has been difficult to construct high-density genetic maps from intra-varietal crosses to facilitate genetic mapping and QTL analysis of important traits (disease resistance, fruit yield etc.). To date, the most saturated intra-varietal map is constructed by Yang *et al.* with 735 SSRs and a mean marker interval of 0.96 cM [11]. The SNP map developed herein contained 1,800 SNPs and a majority of them were anchored to ‘9930’ draft genome scaffolds (Table 2). As compared with the 735-SSR map [11], the number of mapped loci, marker density (from 0.96 cM to 0.50 cM), and total map length (706.7 cM vs. 890.79 cM) is significantly improved in the SNP genetic map. The marker number in this individual map is also an increase in comparison with the two consensus maps by Zhang *et al.* [53] and Yang *et al.* [26]. We anchored 183 scaffolds of ‘9930’ genome scaffolds covering 168.9 Mb of the cucumber genome, which is exceptionally not an increase contrasted to the 735-point (237 scaffolds, 193.3 Mb), and two integrated maps (1369-point map, 172.5 Mb; 1681-point map, 275 scaffolds and 202.3 Mb). This suggests more SNP markers are required to anchor scaffolds and cover the entire physical distance of cucumber genome. Marker locations on the genetic map and in the scaffolds could infer the quality of both the genetic map and the ‘9930’genome scaffolds. It was demonstrated in several studies that there are mis-assembled scaffolds in the published cucumber draft assemblies [11, 21, 54, 55]. In our study, two mis-assembled scaffolds in chromosome 5 were verified by single-copy gene FISH experiment. This indicated that the SNP-based genetic map constructed herein could be applied to detect mis-assembled scaffolds. However, further efforts are needed to address all the scaffold positions as well as improve quality of the SNP map.

### QTL analysis of fruit-related traits

This study is the first attempt to conduct QTL analysis using a NGS-derived genetic map in cucumber. Fruit weight and length are two traits that have direct impacts on field yield of cucumber, thus are important for cultivar improvements. We identified 9 QTLs for fruit weight and length of mature and immature fruits on three linkage groups. Three QTLs were detected on LG1; one was on LG6, and five on LG3 for fruit yield traits. Two pairs of QTLs for mature fruit length and immature fruit length shared similar location intervals, *fl1.1* and *mfl1.1*, *fl3.1* and *mfl3.1*. QTLs located in two adjacent marker intervals might take function as one locus. In the previous studies, QTLs controlling fruit length were detected on all of the seven chromosomes/linkage groups using F_2_ or RIL populations based on different types of markers (i.e. SRAP, SSR, and morphological markers). For example, Cheng *et al.* [42] and Miao *et al.* [43] identified five QTLs (on LG1, LG4 and LG6) and three QTLs (on LG5 and LG6) that explained 7.1% ~ 14.1% of the phenotypic variation. Among 8 QTL s detected by Yuan *et al.* [40], the QTL on LG4 explained 23.32% of the fruit length variance, which was a dominant QTL. Both of the two predominant QTLs on LG3 in the present study explained ~ 40% of the observed variance. We identified two QTLs for immature fruit weight on LG3, which shared one linkage group with the report by Yuan *et al.* [40], whereas no common LGs with the results from Chen *et al.* [41]. The differences in QTL number, position and phenotypic variance explained might be attributed to different mapping populations (genotypes, population size etc.) and environment effects [6, 8, 39, 40, 56].

Generally, the detected QTL regions in most studies were covered by two markers. Nevertheless the chromosomal interval for QTLs detected in the present study could cover up to 20 SNPs. For example, the chromosomal region accounted for *fl6.1* on LG6 (57.0 ~ 66.7 cM) covered 25 SNPs (Additional file 5 and Additional file 6, Additional file 1: Figure S2). This increase in mapped markers is likely to facilitate fine mapping of these QTLs in further studies.

## Conclusion

We generated 10.76 Gb raw data, 75,024,043 pair-end reads and 52,684 high-quality SLAFs using SLAF sequencing. A high-resolution genetic map for cucumber was constructed containing 1,800 SNPs with a total genetic length of 890.79 cM. The mean distance between adjacent markers was 0.50 cM. This SNP-based genetic map could be applied to anchor and orient draft genome scaffolds. Physically, 183 scaffolds from the ‘9930’ draft genome assemblies were anchored. Although number of anchored scaffolds was not really an increase compared to existing cucumber consensus maps, the SNP map derived from *de novo* sequencing of a reduced representation genome could still be used to evaluate the quality of draft scaffold assemblies. Two mis-assembled scaffold were verified by FISH with newly developed single-copy genes. We further applied this linkage map to detect QTLs controlling fruit-related traits. Nine QTLs were identified for fruit length and weight. To date, this study is the first report of large-scale SNP identification and genotyping in cucumber. The 1,800-SNP map constructed herein would likely facilitate fine genetic mapping of fruit-related QTLs and orientation of draft genome scaffolds.

## Methods

### Plant materials and phenotypic evaluation

Cucumber inbred lines ‘CC3’ and ‘NC76’ were used as female parent and male parent, respectively. The fruits of CC3 female plant were characterized as non-netting, long fruits (20 ~ 30 cm) with smooth skin, white spines, and green/creamy mature fruit color. NC76 had short fruit (7 ~ 10 cm) with coarse skin, heavy netting, black spines and red mature fruit color. A population of F_2_ with 148 individuals was produced from a single F_1_ plant, and was then self-pollinated to generate the F_3_ families. Considering that plants might die of disease and other factors, 12 plants were randomly selected from the F_3_ families and planted. Phenotypic data from 10 healthy plants were used to represent each F_2_ individual. Fruit-related traits (immature and mature fruit length, and immature fruit weight) for each plant of the F2 population and F_3_ family were evaluated for genetic mapping and QTL analysis. The fruit-related traits were measured according to the standards published by Yuan *et al.* [8].

Two parental lines (CC3 and NC76), derived F_1_, F_2_, and F_3_families were grown in a greenhouse at Jiangpu Cucumber Research Station of Nanjing Agricultural University (JCRSNAU), Nanjing, China. The soil media was 25% peat + 25% cinder + 50% perlite. The F_2_ population was planted in March 2012, and evaluations were conducted on both immature (10 days after pollination) and mature (30 ~ 45 days after pollination) cucumber fruits. The evaluated traits were fruit length (*fl* for immature fruits and *mfl* for mature fruit, cm; from fruit apex to the pedicel attachment), fruit weight (*fw for* immature fruit weight, g). These traits were also measured in F3 family fruit in the spring of 2013 under the same growing conditions as F_2_ population. Individual plants were spaced 40 cm apart in rows placed 60 cm apart. All measurements were taken on individual plants and averaged within each F3 family. Data were analyzed with analysis of variance and partial correlations using Microsoft Excel 2000 [57].

### DNA extraction

Young healthy leaves from two parents as well as 148 F2 individuals were collected and frozen in liquid nitrogen, then transferred to a -70°C freezer. Total genomic DNA was extracted from each leaf sample following the cetyltrimethyl ammonium bromide (CTAB) method described by Murray *et al.* [58]. The concentration and quality of extracted DNA were examined by electrophoresis in 1% agarosegels with a standard lambda DNA, and anND-1000 spectrophotometer (NanoDrop, Wilmington, DE, USA).

### SLAF library preparation and sequencing

A pre-experiment was designed to evaluate the enzymes and sizes of restriction fragments to generate large number and high-quality SLAFs. The uniformity of sequencing depth of different fragments was controlled by selecting a tight length range (about 30 ~ 50 bp) and performing a pilot PCR amplification, only fragments with similar amplification features on the gel were maintained. SLAF library construction was performed following the procedures as described by Sun *et al*. [36] and Zhang *et al*. [37]. After the digestion (enzyme *Mse*Iwas used), polymerase chain reactions (PCR) and purification of genomic DNA, fragments(with indices and adaptors) of 330 ~ 380 bp were isolated using Gel Extraction Kit (Qiagen) and subjected to PCR amplification (barcode2 was added). The products were gel purified, and DNA fragments (SLAFs) of 330 ~ 380 bp were recovered and diluted for sequencing. Pair-end sequencing was performed on Illumina High-seq 2000 sequencing platform (Illumina, Inc; San Diego, CA, U.S.) at Biomarker Technologies Corporation in Beijing (http://Biomarker.com.cn/). Each cycle was real-time monitored during sequencing, we also calculated the ratio of high quality reads with quality scores higher than Q20 (a quality score of 20; indicating a 1% chance of an error, and thus 99% confidence) in the raw reads and guanine-citosine (GC) content as standards to control the quality.

### SLAF-seq data analysis and genotyping

SLAF-seq data was operated using the software developed by Sun *et al*. [36]. All pair-end reads generated from SLAF-seq (with clear index information) were clustered according to sequence similarity, which could be inferred from one-to-one alignment by BLAT (-tileSize = 10 -stepSize = 5). Identical reads were merged to avoid repeat computing requirements, and sequences with over 90% similarity were grouped into one SLAF locus as described [36]. Minor allele frequency (MAF) evaluation was performed to define alleles in each SLAF. In the mapping populations of diploid cucumber, one locus can contain no more than four SLAF tags, thus the groups with over four tags were considered as repetitive SLAFs and excluded. In this study, SLAFs with a sequence depth of less than 148 were defined as low-depth SLAFs. Polymorphic SLAFs, which referred to SLAFs that contained 2 ~ 4 tags, were considered as potential markers. Those polymorphic SLAF markers were then assorted into eight segregation patterns as following: ab × cd, ef × eg, hk × hk, lm × ll, nn × np, aa × bb, ab × cc and cc × ab. Since the F_2_ mapping population was derived from two homozygous cucumber inbred lines with a genotype of aa or bb, therefore only the SLAF markers which had segregation patterns of aa × bb were used in map construction.

### Genetic map construction and QTL analysis

Genotype data from the F_2_ mapping population was used to perform linkage analysis using JoinMap 4.0 software [59]. A logarithm of odds (LOD) threshold between 5 and 10 was adopted as indicator for clustering analysis with single linkage method. We employed the Kosambi mapping function to convert recombination percentages to genetic distance. Newly developed SNPs were named by Marker_. The SNP markers were ordered and grouped into seven linkage groups according to genomic information of cucumber. For all SNP markers, corresponding sequence for each SNP was BLAST against the ‘9930’ genome scaffold assemblies version 2.0 [22] (http://cucumber.genomics.org.cn) to anchor and orient scaffolds.

QTL analysis was conducted with MapQTL 4.0 software [60] using both interval mapping method and multiple-QTL model mapping (MQM) methods as described [61, 62]. Composite interval mapping (CIM) was adopted with a walking speed of 1 cM [63]. Two-LOD support intervals were constructed as 95% confidence intervals [64]. The significance of each QTL interval was tested by a likelihood-ratio statistic (LOD). The threshold of the LOD score for significance (P = 0.05) was determined using 1,000 permutations. Calculation of the percentage of phenotypic variance explained by each QTL (Expl.%) was done in MapQTL4.0 based on the population variance found within the segregation population.

### Cytological validation with fluorescence *in situ*hybridization

Pachytene chromosome preparations and slide treatment were performed as described previously [24, 54]. Design and isolation of single-copy gene probes were conducted followed the description by Lou *et al.* [54]. Two single-copy genes, Csa015370 and Csa019548 were selected randomly from scaffold000063_5 and scaffold000063_1, respectively. Flanking sequences of the genes were adopted for primer design to obtain amplification products > 2000 bp. The primers were designed using PRIMER PREMIER 5.0 software. The primer pairs 5′-TAACGCACTCAGTCACTCATTG-3′, 5′-CTTCTGCTTCGCTTCATCC-3′ and 5′-TCCCAACTCCCCACCACTT-3′, 5′-CGTAATCCTCCATCTTTTATCCTCT-3′ were used for amplifying two genes above, respectively. Amplified PCR products were resolved on 1% agarose gels (BIO-WEST) for 30 min at 120 V, and stained with ethidium bromide. Products of the expectedsize were cut from the gel and purified using a gel recovery kit (Promega). The single-copy genes from the purified PCR products were used for FISH. Csa015370 was labeled with digoxigenin-11-dUTP, and Csa019548 was labeled withbiotin-16-UTP. They were detected using a fluorescein isothiocyanate-conjugated antibiotin antibody and arhodamine-conjugated anti-digoxigenin antibody (Roche Diagnos-tics), respectively. The FISH experiment was performed as previously described [55]. Images were captured using a SENSYS CCD camera attached to an Olympus BX51 microscope and processed using ADOBE PHOTOSHOP 5.0 (Adobe Systems). The CCD camera was controlled using FISH view 5.5 software (Applied SpectralImagingInc).

### Availability of supporting data

Raw sequence reads have been deposited in NCBI’s Sequence Read Archive (Study accession: SRP050237). Besides, sequence information of all SNPs is included as additional files.

## Electronic supplementary material

Additional file 1: Figure S1: QTL analysis of immature fruit length on LG 3. **Figure S2.** QTL analysis of immature fruit length on LG 6. **Figure S3.** QTL analysis of fruit weight on LG 3. **Figure S4.** QTL analysis of mature fruit length on LG1. **Figure S5.** Distribution of DNA fragments digested by enzyme on the genome. (PDF 704 KB)

Additional file 2: Total S1: Total SLAFs. **Table S2.** Sequence information of mapped SNP markers. **Table S3.** Detailed marker information in QTL regions. **Table S4.** Genotype encoding rule including eight segregation patterns. (XLSX 2 MB)

Additional file 3: **A high-density cucumber genetic map composing of SNPs.** (PDF 855 KB)

Additional file 4: **Detailed SNP marker information for each of the seven linkage groups.** (XLSX 93 KB)

Additional file 5: **Heat maps for seven cucumber linkage groups indicating map quality.** (PDF 553 KB)

Additional file 6: **Phenotypic evaluation of fruit-related traits for F**_**2**_ **and F**_**3**_ **.** (XLSX 29 KB)

## Abbreviations

SLAF: Specific-length amplified fragments
SNP: Single-nucleotide polymorphism
SLAF-seq: Specific-length amplified fragments sequencing
MAS: Marker-assistant selection
LG: Linkage group
QTLs: Quantitative trait loci
RAPDs: Random amplified polymorphic DNA
AFLPs: Amplified fragment length polymorphisms
SSRs: Simple sequence repeats
RILs: Recombination inbred lines
RRL: Reduced-representation library
RAD: Restriction-site associated DNA
NGS: Next-generation sequencing
SRAP: Sequence-related amplified polymorphism
FISH: Fluorescence *in situ* hybridization.
