# Supplementary material for: An SNP-based saturated genetic map and QTL analysis of fruit-related traits in cucumber using specific-length amplified fragment (SLAF) sequencing
Source: BMC Genomics. 2014 Dec 22;15(1):1158. doi: 10.1186/1471-2164-15-1158 (PMC4367881; doi:10.1186/1471-2164-15-1158)
Supplement: Supplementary file 1 — Additional file 1: Figure S1: QTL analysis of immature fruit length on LG 3. Figure S2. QTL analysis of immature fruit length on LG 6. Figure S3. QTL analysis of fruit weight on LG 3. Figure S4. QTL analysis of mature fruit length on LG1. Figure S5. Distribution of DNA fragments digested by enzyme on the genome. (PDF 704 KB) [file 12864_2014_6913_MOESM1_ESM.pdf]

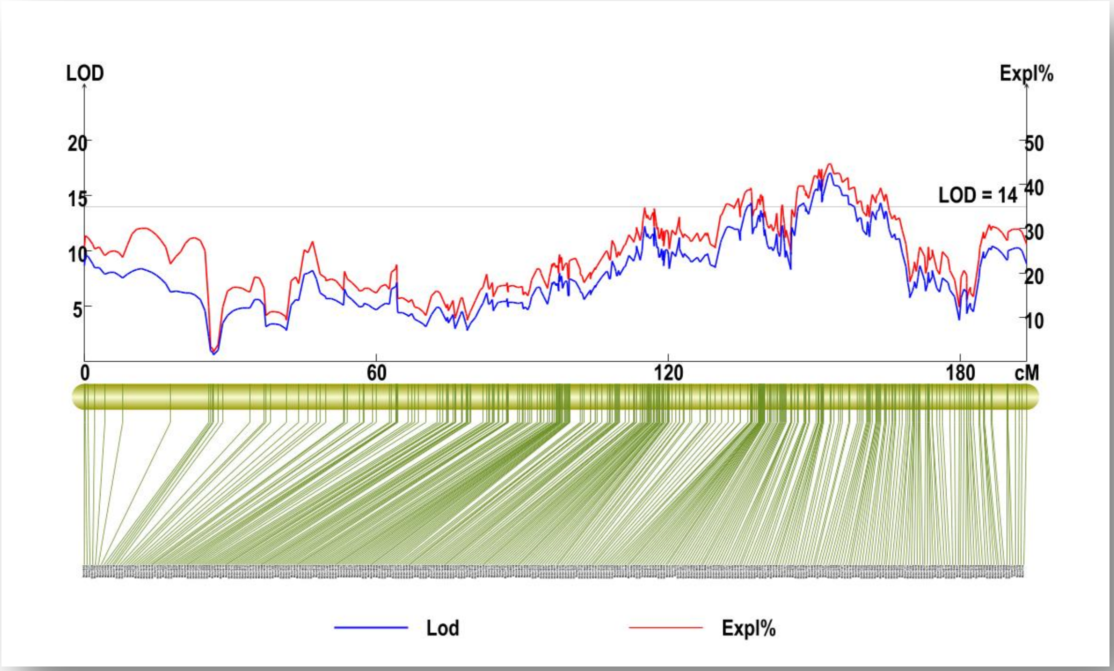

Figure S1. QTL analysis of immature fruit length on LG 3

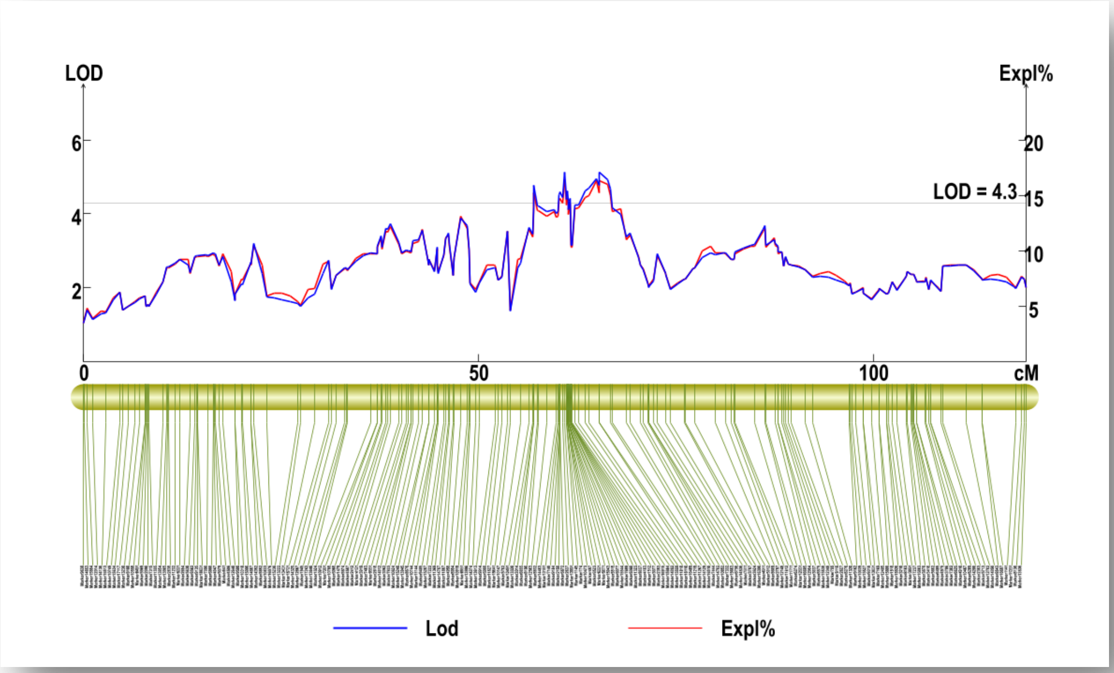

Figure S2. QTL analysis of immature fruit length on LG 6

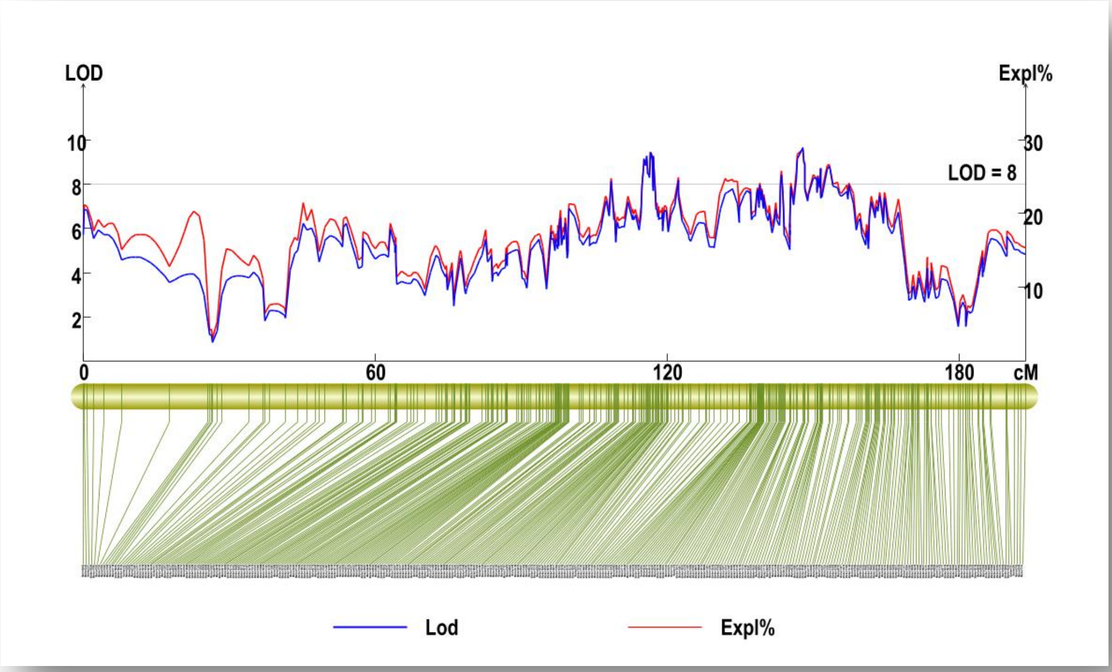

Figure S3. QTL analysis of fruit weight on LG 3

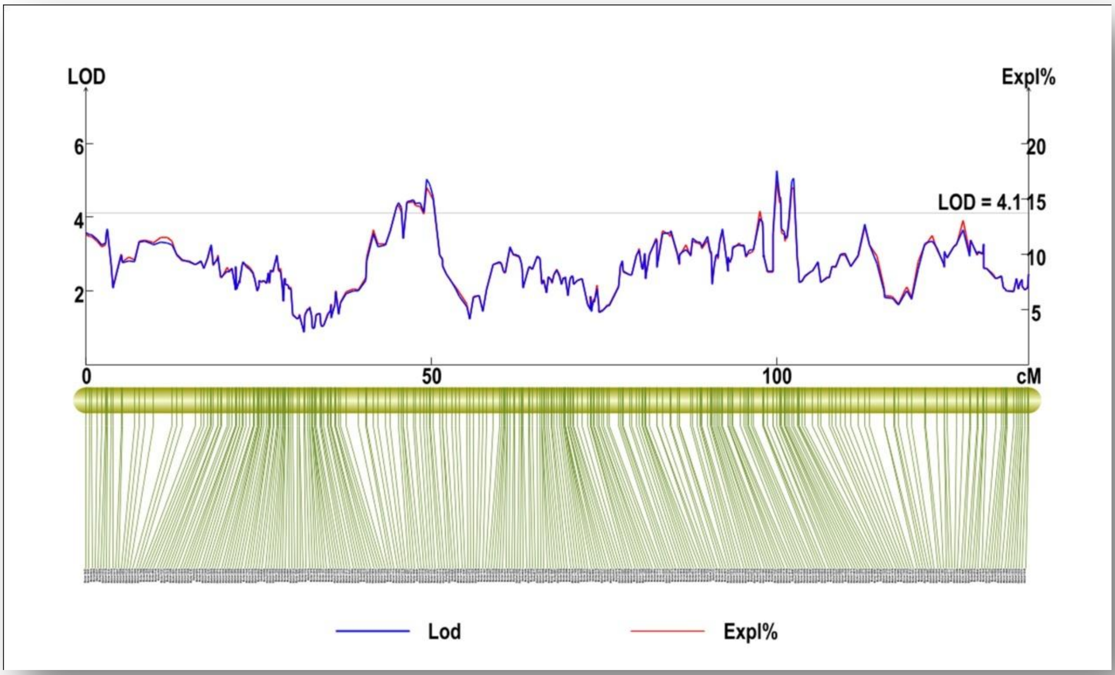

Figure S4. QTL analysis of mature fruit length on LG1

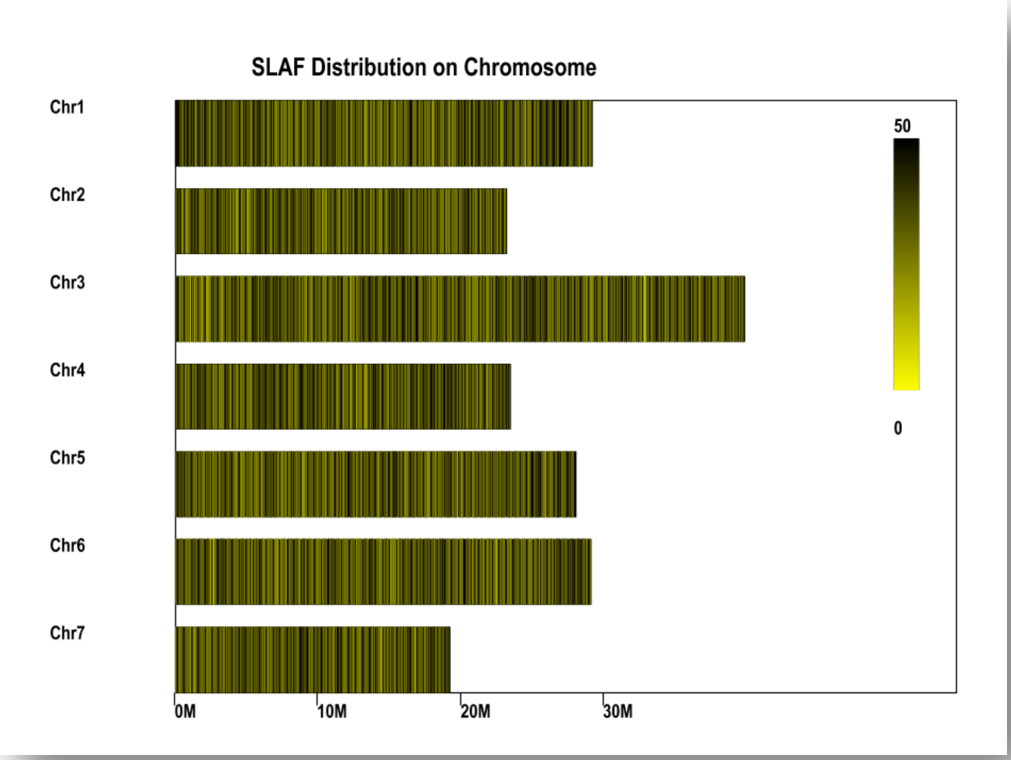

Figure S5. Distribution of DNA fragments digested by enzyme on the genome
