# Supplementary figures and images for: An SNP-based saturated genetic map and QTL analysis of fruit-related traits in cucumber using specific-length amplified fragment (SLAF) sequencing
Source: BMC Genomics. 2014 Dec 22;15(1):1158. doi: 10.1186/1471-2164-15-1158 (PMC4367881; doi:10.1186/1471-2164-15-1158)

A high-density cucumber genetic map composing of SNPs

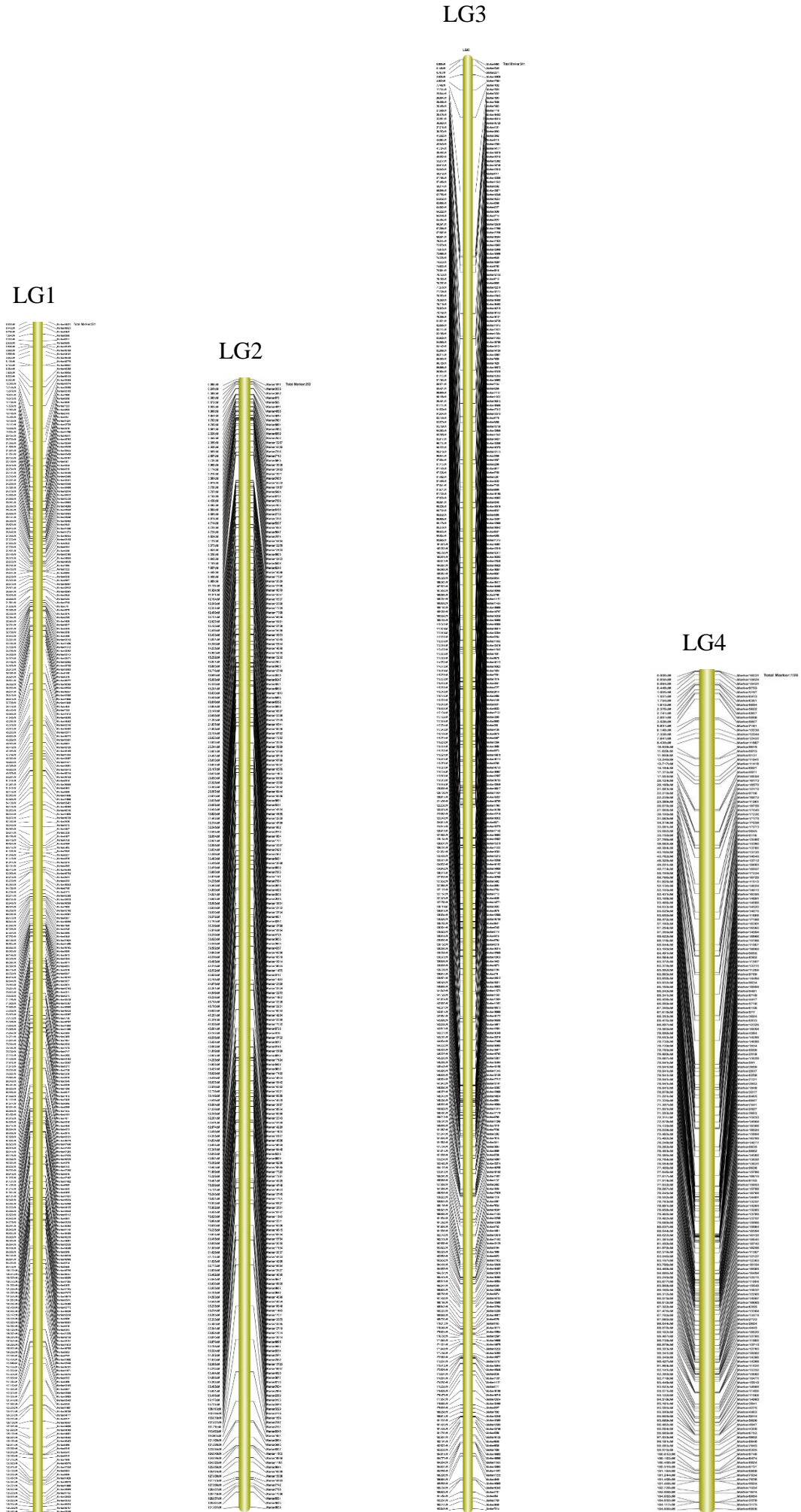

LG5

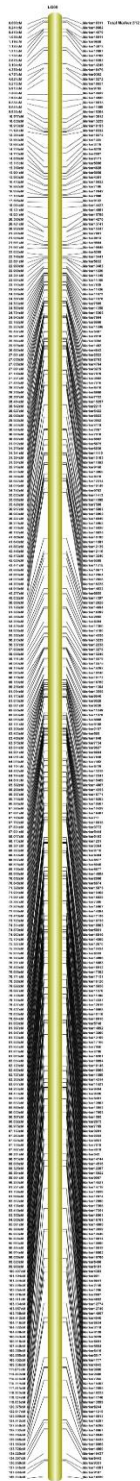

LG6

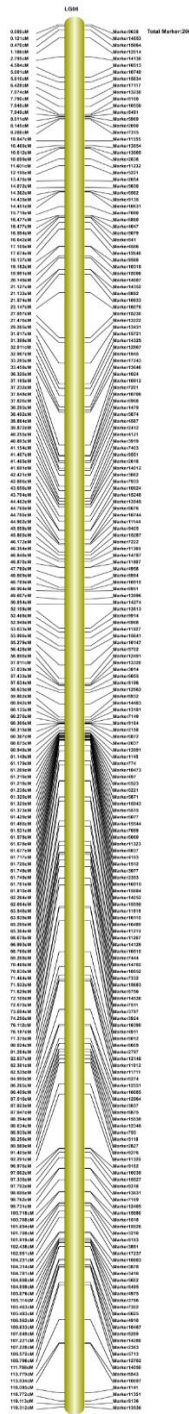

LG7

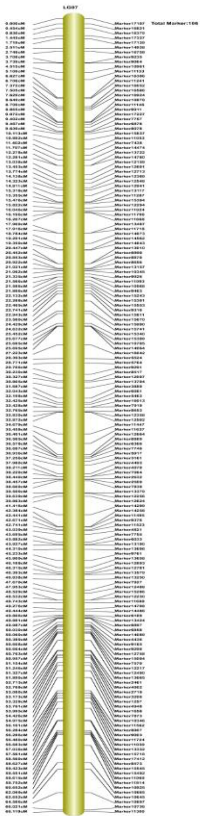

Supplement: Supplementary file 3 — Additional file 3: A high-density cucumber genetic map composing of SNPs. (PDF 855 KB) [file 12864_2014_6913_MOESM3_ESM.pdf]
