# Supplementary material for: An SNP-based saturated genetic map and QTL analysis of fruit-related traits in cucumber using specific-length amplified fragment (SLAF) sequencing
Source: BMC Genomics. 2014 Dec 22;15(1):1158. doi: 10.1186/1471-2164-15-1158 (PMC4367881; doi:10.1186/1471-2164-15-1158)
Supplement: Supplementary file 5 — Additional file 5: Heat maps for seven cucumber linkage groups indicating map quality. (PDF 553 KB) [file 12864_2014_6913_MOESM5_ESM.pdf]

Heat maps for seven cucumber linkage groups indicating map quality

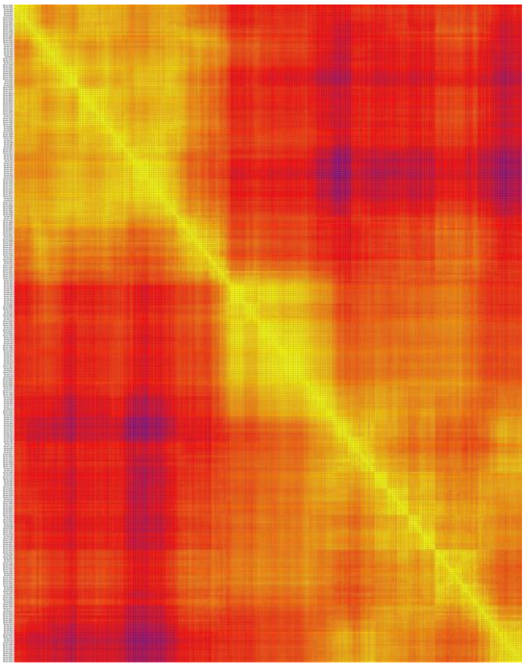

LG1.heatMap

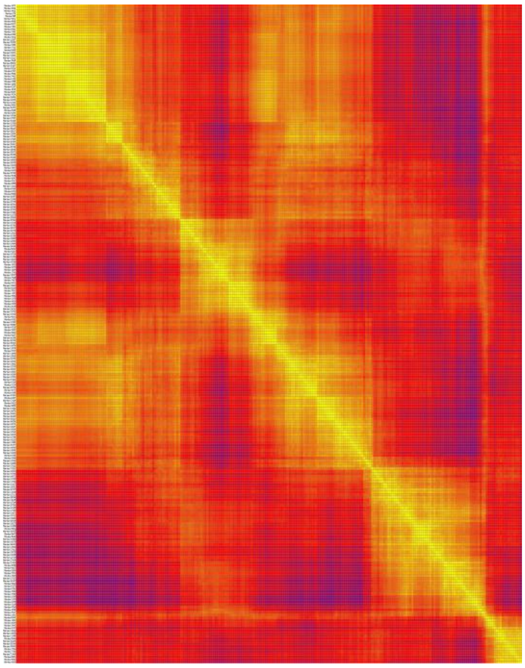

LG2.heatMap

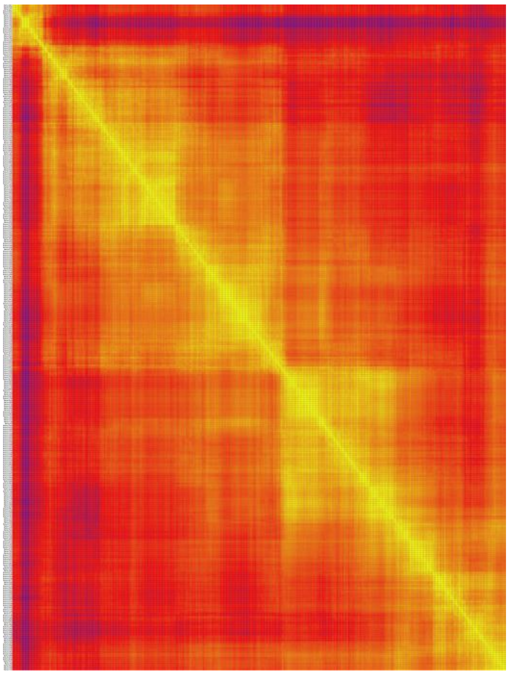

LG3.heatMap

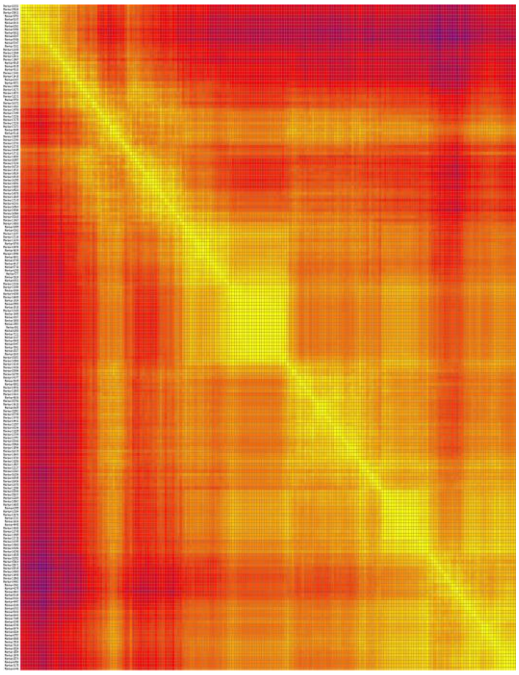

LG3.heatMap

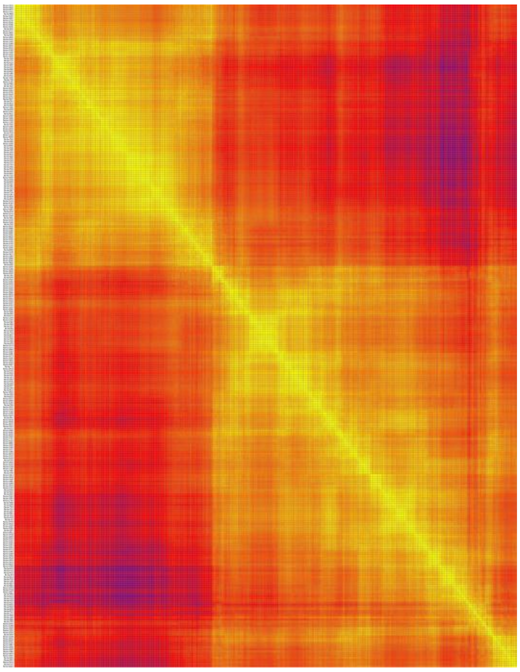
LG5.heatMap

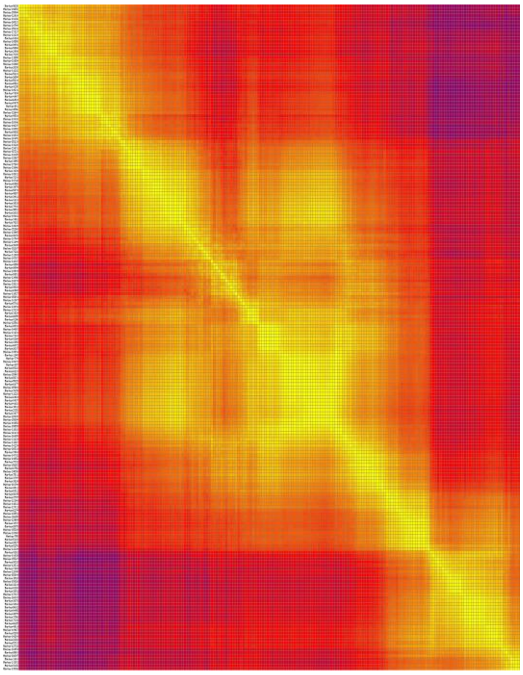
LG6.heatMap

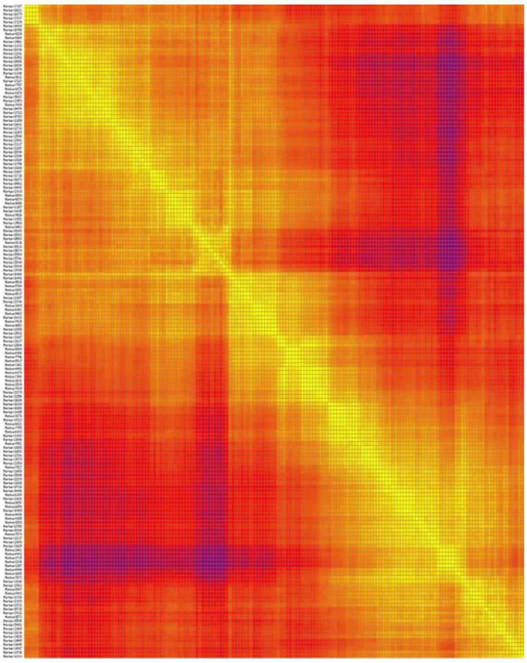
LG7.HeatMap
